# Expression regulation of bacterial lipase genes: a review

**DOI:** 10.3389/fmicb.2025.1592059

**Published:** 2025-05-21

**Authors:** Dai-ming Zha, Yun-jun Yan

**Affiliations:** ^1^School of Pharmacy and Life Sciences, Jiujiang University, Jiujiang, China; ^2^Key Laboratory of Molecular Biophysics, Ministry of Education, College of Life Science and Technology, Huazhong University of Science and Technology, Wuhan, China

**Keywords:** lipase, expression regulation, Gac/Rsm system, QS system, two-component system

## Abstract

Microbial lipases constitute the primary source of commercialized and industrial lipases, and they are extensively utilized across numerous industrial sectors. Compared to fungal lipases, bacterial lipases catalyze a broader spectrum of reactions with higher activity, enhanced stability, and improved stress resistance. Among them, lipases from *Pseudomonas* and *Burkholderia cepacia* are among the most widely employed microbial lipases. Furthermore, bacterial extracellular lipases act as crucial virulence factors, playing a significant role in the pathogenesis of bacteria. However, the production of bacterial lipases is typically low, rendering them expensive in the market and insufficient to meet the substantial demand for industrial production. To achieve large-scale production of bacterial lipases, stable and efficient homologous expression has proven to be an effective strategy. However, elucidating how bacterial lipase genes are regulated is the initial step for developing stable and efficient homologous expression, and a pressing scientific challenge. To date, the regulatory mechanisms governing the expression of bacterial lipase genes remain unclear, significantly impeding the construction of robust and high-yield homologous expression systems. Concurrently, understanding these regulatory mechanisms can facilitate early diagnosis of lipase-related pathogenic bacterial infections, and aid in the development of novel antibacterial drugs. In this review, we summarized the advancements in understanding the expression regulation of bacterial lipase genes, including direct regulators, the quorum sensing (QS) system, the Gac/Rsm system and its related regulators, as well as other regulators. Additionally, based on our ongoing research, we also discussed potential research directions in this field, aiming to provide valuable insights for the construction of homologous expression systems with high-yield lipases.

## 1 Introduction

Lipases (triacylglycerol acylhydrolases, EC 3.1.1.3), belonging to the *α*/*β* hydrolase superfamily, are ubiquitous in various animals, plants and microorganisms. They can catalyze the hydrolysis of long-chain acylglycerols at the oil–water interface, and also various synthetic reactions in micro-aqueous or non-aqueous phases, such as transesterification, esterification, aminolysis and alcoholysis (Jaeger et al., 1999; Ali et al., 2023; Kim et al., 2023; Zhao et al., 2024). They are the third most important industrial enzymes after proteases and amylases (Filho et al., 2019). Microbial lipases are the primary source of commercialized and industrial lipases, widely used in numerous fields such as food, beverages, oils and fats, detergents, feed, textiles, leather, novel materials, fine chemicals, pharmaceuticals, cosmetics, papermaking, environmental treatment, bioenergy, etc. (Jaeger et al., 1999; Enespa and Singh, 2023; Ali et al., 2023; Kim et al., 2023). Compared to fungal lipases, bacterial lipases catalyze a wider range of reactions with higher activity, better stability and stress resistance. Among them, lipases form *Pseudomonas* and *Burkholderia cepacia* are one of the most widely used microbial lipases (Arpigny and Jaeger, 1999; Jaeger et al., 1999; Gupta et al., 2004; Sánchez et al., 2018). In addition, bacterial extracellular lipases are also important virulence factors that play a significant role in the pathogenesis of bacteria (Stehr et al., 2003).

However, the production of bacterial lipases is generally low, and various strategies such as conventional breeding, fermentation condition optimization and heterologous expression have failed to effectively address this issue. As a result, bacterial lipases are costly and hard to meet the large industrial demand. Rosenau and Jaeger proposed that the large-scale production of bacterial lipases could be achieved through the following three steps: elucidating the molecular mechanisms on the expression regulation of lipase genes, constructing genetically engineered strains for stable and efficient homologous expression of lipases, and optimizing the fermentation conditions for these genetically engineered strains (Rosenau and Jaeger, 2000). However, to date, the above regulatory mechanisms remain unclear, which significantly hinders the construction of these genetically engineered strains. Additionally, elucidating these mechanisms can also facilitate early diagnosis of lipase-related pathogenic bacterial infections and the development of new antibacterial drugs. Therefore, studying the above mechanisms holds significant theoretical and practical importance.

**Figure 1:**
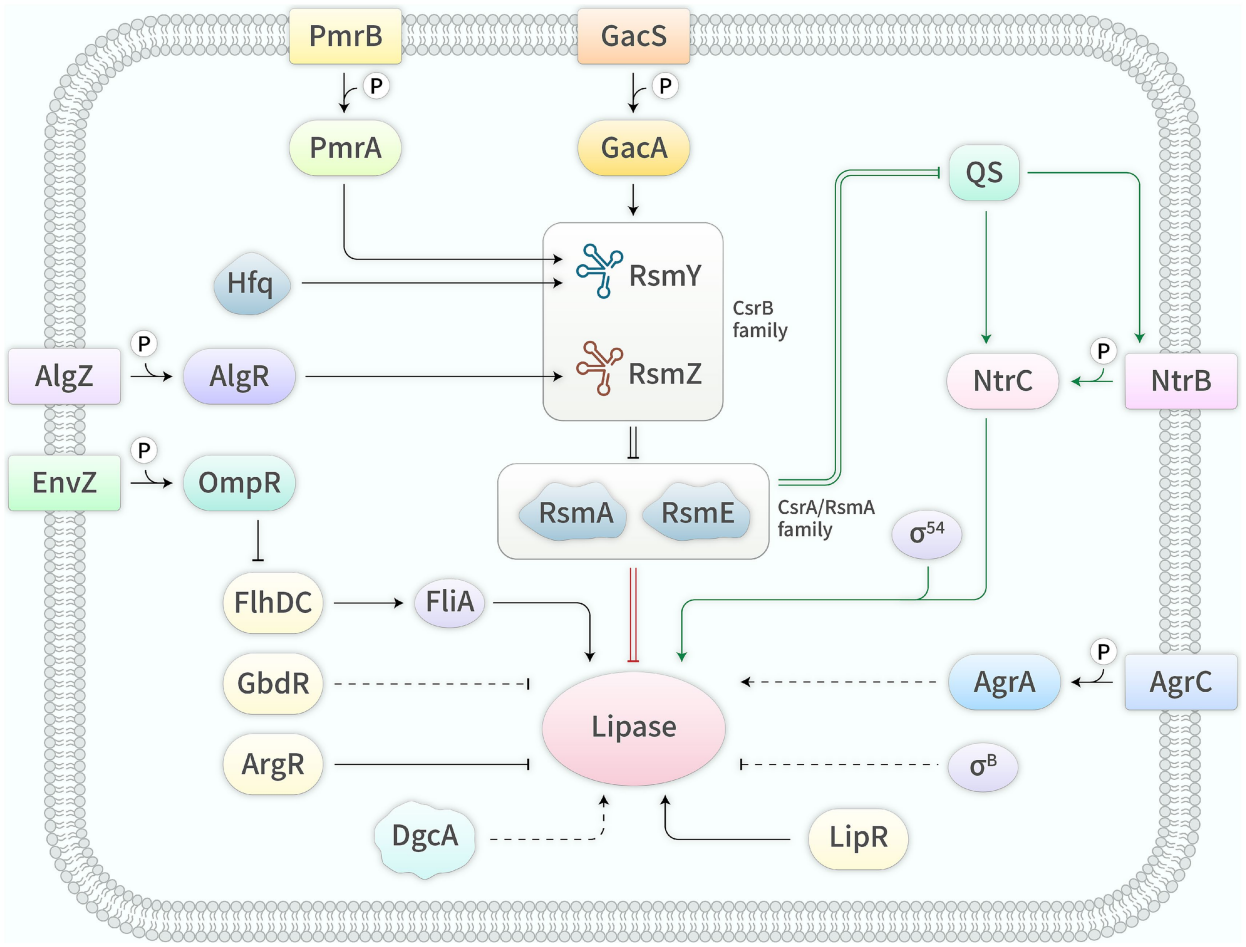
Regulators and their mechanisms in the expression of bacterial lipase genes. →, activation; —|, inhibition; =, interaction; —, known mechanism; ┄, unknown mechanism.

Currently, research hotspots in the field of lipases mainly include gene resource mining, enzymatic property characterization, immobilization technology and industrial applications. However, research progress in gene expression regulation has been slow, which is unfavorable for elucidating the regulatory mechanisms and severely obstructs the construction of genetically engineered strains. Up to now, the expression of bacterial lipase genes is primarily regulated by direct regulators, the QS system, the Gac/Rsm system and its related regulators, as well as other regulators (Figure 1 and Table 1). The regulatory mechanisms of these regulators are detailed below.

**Table 1:** Regulators and their mechanisms in the expression of bacterial lipase genes.

Regulator	Species	Effect	Mechanism	References
Direct regulators				
NtrB/C superfamily	*P. aeruginosa*, *P. alcaligenes*	Activation	Directly activating the transcription of lipase genes by binding to the UAS.	Jaeger et al. (1999), Rosenau and Jaeger (2000), Krzeslak et al. (2008), and Krzeslak et al. (2012)
σ^54^	*P. aeruginosa*, *P. alcaligenes*	Activation	Assisting NtrC superfamily in activating the transcription of lipase genes.	Jaeger et al. (1999), Rosenau and Jaeger (2000), Cox et al. (2001), and Krzeslak et al. (2012)
LipR	*S. exfoliatus*, *S. coelicolor*	Activation	Directly activating the transcription of *lipAR*-operon by binding to conserved sequences upstream of the −35 region.	Servin-Gonzalez et al. (1997) and Valdez et al. (1999)
FliA (σ^28^)	*X. nematophila*	Activation	Directly enhancing *xlpA* transcription by binding to the consensus binding sequence contained in the *xlpA* promoter.	Park and Forst (2006)
RsmE	*P. protegens*	Inhibition	Directly inhibiting the translation of *lipA* through binding to the SD sequence of *lipA* mRNA.	Zha et al. (2014)
ArgR	*P. aeruginosa*, *P. protegens*	Inhibition	Restraining *lipA* transcription by an unknown mechanism in *P. aeruginosa*, and also directly repressing *lipA* transcription by binding to the ArgR binding site located in the *lipA* promoter in *P. protegens*.	Yang and Lu (2007) and Ying et al. (2019)
**QS system**				
QS	*P. aeruginosa*	Activation	Transcriptionally activating *lipA* mediated by directly enhancing the transcription of *cbrA/B*.	Jaeger et al. (1999), Rosenau and Jaeger (2000), Duan and Surette (2007), Boyer and Wisniewski-Dye (2009), and Williams and Cámara (2009)
**Gac/Rsm system**				
Gac/Rsm	*P. aeruginosa*, *P. protegens*	Activation and inhibition	Primarily promoting *lipA* transcription through the Gac/RsmA/QS/CbrA/B/LipA pathway, and secondarily inhibiting *lipA* transcription through the Gac/RsmA/unknown regulator(s)/LipA pathway in *P. aeruginosa*; primarily and directly activating *lipA* translation through the Gac/RsmE/LipA pathway, and secondarily and indirectly activating *lipA* transcription through the Gac/RsmA/unknown regulator(s)/LipA pathway in *P. protegens*.	Reimmann et al. (1997), Jaeger et al. (1999), Rosenau and Jaeger (2000), Heurlier et al. (2004), Williams and Cámara (2009), Lalaouna et al. (2012), and Zha et al. (2014)
RsmA	*P. aeruginosa*, *P. protegens*	Activation and inhibition	Primarily inhibiting *lipA* transcription through the RsmA/QS/CbrA/B/LipA pathway by binding to the 5’UTR of *lasI* and *rhlI* mRNAs, and secondarily promoting *lipA* transcription by an unknown mechanism in *P. aeruginosa*; mainly activating *lipA* translation by inhibiting *rsmE* translation and secondarily inhibiting *lipA* transcription by an unknown mechanism in *P. protegens*.	Rosenau and Jaeger (2000), Heurlier et al. (2004), Williams and Cámara (2009), and Zha et al. (2014)
**Regulators mediated by Gac/Rsm system**				
AlgZ/R	*P. protegens*	Activation	Mainly promoting *lipA* transcription by directly binding to the promoter sequence of *rsmZ*, and also enhancing *lipA* translation by an unknown mechanism.	Li et al. (2017)
Hfq	*P. protegens*	Activation	Mainly strengthening *lipA* translation through increasing the transcript of *rsmY* and enhancing the stability of RmsY, and also boosting *lipA* transcription by an unknown mechanism.	Liu et al. (2017)
PmrB/A	*P. aeruginosa*	Activation	Mainly activating *lipA* translation through the PmrB/A/RsmY/RsmA/LipA pathway by directly binding to the promoter sequence of *rsmY*, and also promoting *lipA* transcription by an unknown mechanism.	Liu et al. (2018)
**Other regulators**				
σ^B^	*S. aureus*	Inhibition	Repressing the production of lipase by an unknown mechanism.	Kullik et al. (1998)
AgrC/A	*S. aureus*	Activation	Enhancing the production of lipase by an unknown mechanism.	McNamara and Iandolo (1998)
EnvZ/OmpR	*P. fluorescens*, *X. nematophila*	Inhibition	Inhibiting *lipA* expression by an unknown mechanism in *P. fluorescens*; repressing *xlpA* transcription through the EnvZ/OmpR/FlhDC/FliA/XlpA pathway in *X. nematophila*.	McCarthy et al. (2004) and Park and Forst (2006)
DgcA	*P. aeruginosa*	Activation	Activating *lipA* expression by an unknown mechanism.	Hampel et al. (2014)
GbdR	*P. aeruginosa*	Inhibition	Suppressing *lipA* expression by an unknown mechanism.	Hampel et al. (2014)

## 2 Direct regulators

Several studies have shown that the transcription of bacterial lipase genes is directly regulated by the two-component system NtrB/C superfamily (Jaeger et al., 1999; Rosenau and Jaeger, 2000; Krzeslak et al., 2008; Krzeslak et al., 2012). The NtrB superfamily is a class of sensor kinases that can sense extracellular environmental signals, although the nature of the signaling molecules remains unclear. In contrast, the NtrC superfamily, being a class of response regulators, can bind to the upstream activating sequence (UAS) of the promoter, thereby activating the transcription of lipase genes. Upon sensing environmental signaling molecules, the NtrB superfamily undergoes autophosphorylation and transfers the phosphate group to the NtrC superfamily, then the phosphorylated NtrC superfamily acts as a class of transcriptional activators (Monteagudo-Cascales et al., 2022). To date, two members of this protein superfamily, i.e., CbrA/B in *P. aeruginosa* and LipQ/R in *P. alcaligenes*, have been reported to directly regulate the transcription of lipase genes (Jaeger et al., 1999; Rosenau and Jaeger, 2000; Krzeslak et al., 2008; Abdou et al., 2011; Krzeslak et al., 2012). It is worth noting that the NtrC superfamily requires the assistance of the alternative sigma factor σ^54^ to activate gene transcription, and σ^54^-defective strains produce little or no lipase (Jaeger et al., 1999; Rosenau and Jaeger, 2000; Cox et al., 2001; Krzeslak et al., 2012). Moreover, the lipase operon *lip/lif* from *P. aeruginosa* contains two promoters, P1 and P2, and its transcription initiated by P1 requires σ^54^, while the physiological function of P2 remains unclear and requires further investigation (Jaeger et al., 1999; Rosenau and Jaeger, 2000). Similarly, in *B. glumae*, two putative σ^54^-dependent promoters were identified ahead of the lipase operon *lipAB*, which suggests that the transcription initiation of this operon may involve the NtrC superfamily and σ^54^. Also in this bacterium, a putative cAMP response protein (CRP) binding site was also found upstream of the *lipAB*-operon, implying that *lipAB* transcription is subjected to catabolite repression and induced by the CRP-cAMP complex in the absence of glucose (Beselin, 2005).

Furthermore, in *Streptomyces* the lipase gene *lipA* and its contiguous and downstream gene *lipR*, encoding a transcriptional activator that belongs to the LuxR family, form an operon and their transcription is directly activated by LipR binding to conserved sequences upstream of the −35 region (Servin-Gonzalez et al., 1997; Valdez et al., 1999). In *Xenorhabdus nematophila*, the flagella sigma factor, FliA (σ^28^), directly enhances the transcription of the lipase gene *xlpA* by binding to the consensus binding sequence contained in the *xlpA* promoter (Park and Forst, 2006). In *P. protegens*, RsmE, a member of the CsrA/RsmA family, was reported to directly inhibit *lipA* translation through binding to the ACAAGGAUGU sequence overlapping the Shine-Dalgarno (SD) sequence of *lipA* mRNA, thereby hindering the access of the 30S ribosomal subunit to the SD sequence (Zha et al., 2014). ArgR, a transcriptional regulator belonging to the AraC/XylS family, plays a key role in arginine metabolism regulation, and in *P. protegens* it has also been confirmed to directly repress *lipA* transcription by binding to the ArgR binding site (TGTCGCCAAAGCGTCATGGGG) located in the *lipA* promoter to produce steric hindrance. In addition, *lipA* expression in both wild-type and *argR* mutant is inhibited by arginine, and arginine exhibits a synergistic inhibitory effect with ArgR (Ying et al., 2019). Similarly, ArgR in *P. aeruginosa* was reported to also restrain *lipA* transcription, and deletion of *argR* significantly increases *lipA* transcript by up to 2.3 times, meanwhile the addition of arginine further enhances this effect, increasing *lipA* transcript by 9 times. The specific mechanism underlying this regulation requires further investigation (Yang and Lu, 2007). The differential regulatory mechanism of ArgR between *P. protegens* and *P. aeruginosa* may result from variations in the promoter sequences of their respective target genes.

## 3 QS system

Numerous studies have demonstrated that the expression of bacterial lipase genes exhibits cell density-dependence, known as QS (Jaeger et al., 1999; Lewenza et al., 1999; Rosenau and Jaeger, 2000; Christensen et al., 2003; Devescovi et al., 2007; Su et al., 2019). QS is a process by which bacteria communicate using self-produced autoinducers as signaling molecules. The QS system is involved in regulating numerous biological functions, including the synthesis of various enzymes and antibiotics, the production of virulence factors, the synthesis of extracellular polysaccharides, and the formation of biofilms and spores (Miller and Bassler, 2001; Boyer and Wisniewski-Dye, 2009; Juszczuk-Kubiak, 2024). To date, the molecular mechanism by which the QS system activates the expression of lipase genes has only been elucidated in *P. aeruginosa*, where it transcriptionally activates *lipA* mediated by directly enhancing the transcription of *cbrA/B* (Jaeger et al., 1999; Rosenau and Jaeger, 2000; Duan and Surette, 2007; Boyer and Wisniewski-Dye, 2009; Williams and Cámara, 2009). In the QS system, bacteria sense population density by producing diffusible small molecule substances known as autoinducers. Most Gram-positive bacteria use small peptides as QS signal molecules, while Gram-negative bacteria utilize various small molecule substances as QS signal molecules, with acyl-homoserine lactones (acyl-HSLs) being the most representative (Lewenza et al., 1999; Juszczuk-Kubiak, 2024). The majority of Gram-negative bacteria employ acyl-HSLs as their QS signal molecules, which are produced by LuxI-type signal synthases and gradually accumulate as the population density increases. When the concentration reaches a certain threshold, they bind to the LuxR-type receptors, enabling the latter to function as transcription activators (Williams and Cámara, 2009; Parsek and Greenberg, 2000; Majdura et al., 2023).

The acyl-HSLs mediated QS system is considered the classical QS system, with the QS system of *P. aeruginosa* being the most representative. It possesses two acyl-HSL signaling systems, namely the Las and Rhl systems. The Las system includes LasI, the signal synthase that produces N-(3-oxo-dodecanoyl)-homoserine lactone (3-oxo-C12-HSL), and LasR, the signal receptor that, upon binding to 3-oxo-C12-HSL, activates the transcription of certain target genes. This activation requires 3-oxo-C12-HSL dependent LasR polymerization. The Rhl system is the second QS system, comprising RhlI, the signal synthase that synthesizes N-butanoyl-homoserine lactone (C4-HSL), and RhlR, the signal receptor that, upon binding to C4-HSL, induces the transcription of certain genes, such as promoting the transcription of *cbrA/B* in the regulation network of lipase gene expression. While the transcriptional activity of RhlR requires C4-HSL, its dimerization does not. LasR and RhlR can also induce the transcription of their respective homologous signal synthase genes, forming a positive feedback loop, the process also known as self-induction, which leads to rapid increase and diffusion of signal molecules. These two QS systems form a cascade relationship, where the Las system activates the Rhl system, meaning that LasR-3-oxo-C12-HSL activates the transcription of *rhlI/R* (Parsek and Greenberg, 2000; Fuqua and Greenberg, 2002; Schuster and Greenberg, 2006; Williams and Cámara, 2009; Majdura et al., 2023).

## 4 Gac/Rsm system

The Gac/Rsm system is highly conserved in Gram-negative bacteria and consists of the two-component system GacS/A, the CsrB family and the CsrA/RsmA family (Lapouge et al., 2008). The GacS/A system has been proven to play a crucial role in the expression regulation of bacterial lipase genes. Deletion of *gacS*, *gacA*, or both *gacS/A* inhibits the expression of lipase genes (Reimmann et al., 1997; Jaeger et al., 1999; Rosenau and Jaeger, 2000; Williams and Cámara, 2009; Lalaouna et al., 2012; Zha et al., 2014). In *P. aeruginosa*, deletion of *gacA* reduces lipase production by 3-fold, whereas overexpression of *gacA* increases lipase production by 1.4-fold (Reimmann et al., 1997). The GacS/A system is composed of the membrane-bound sensor kinase GacS and the response regulator GacA. GacS contains the HAMP domain (histidine kinase, adenylyl cyclase, methyl-accepting protein, and phosphatase), the HisKA domain (histidine kinase), the HATPase-c domain (ATPase), the REC domain (response regulator), and the Hpt domain (phosphotransfer). The HAMP domain is responsible for GacS-GacS interaction, while the HisKA/HATPase-c/REC domains are responsible for GacS-GacA interaction (Workentine et al., 2009; Song et al., 2023). GacS senses unknown signal molecules and undergoes autophosphorylation. It interacts with GacA to transfer the phosphate group to the latter, and phosphorylated GacA functions as a transcription activator, initiating the transcription of the CsrB family of small regulatory RNAs (sRNAs) (Sonnleitner and Haas, 2011; Gallegos et al., 2024). In *P. aeruginosa*, GacA directly regulates only the transcription of two sRNA genes *rsmY* and *rsmZ* (Brencic et al., 2009), while in *P. syringae*, it also directly regulates the transcription of other genes (Cha et al., 2012). The CsrB family of sRNAs are rich in GGA motifs and form multiple stem-loop structures, enabling specific binding to CsrA/RsmA family proteins and alleviating the translational inhibition of target genes (Humair et al., 2010; Sonnleitner and Haas, 2011; Marzi and Romby, 2012). The CsrA/RsmA family is a class of RNA-binding proteins that can bind to multiple specific motifs (often GGA or ANGGA) on the 5′ untranslated region (UTR) of target mRNAs. Since one of these specific motifs is close to or overlaps with the SD sequence, which prevents the recruitment of the 30S ribosomal subunit to the ribosome-binding site (RBS), ultimately inhibiting the translation of the target mRNAs (Sonnleitner and Haas, 2011).

In *P. aeruginosa*, RsmA, a member of the CsrA/RsmA family, which specifically binds to the 5’UTR of *lasI* and *rhlI* mRNAs, leading to their translational inhibition and ultimately resulting in the inhibition of *lipA* expression at the transcriptional level (Rosenau and Jaeger, 2000; Williams and Cámara, 2009). In addition, RsmA also transcriptionally promotes *lipA* expression through another unknown pathway. Deletion of *rsmA* reduces lipase production by 1.9-fold, while overexpression of *rsmZ* lowers lipase production by 5.6-fold but its deletion has almost no effect on lipase production, which may be related to the redundancy of the CsrB family of sRNAs. The stimulative effect of RsmA may be indirect, achieved by repressing inhibitors of *lipA* expression, with the specific inhibitors yet to be further identified (Heurlier et al., 2004). However, in *P. protegens* lacking the classical QS system, RsmA and RsmE, the members of the CsrA/RsmA family, have been shown to regulate *lipA* expression through different pathways. RsmA mainly activates *lipA* translation by inhibiting *rsmE* translation and secondarily inhibits *lipA* transcription through an unknown pathway, while RsmE directly inhibits *lipA* translation by binding to the SD sequence of *lipA* mRNA (Zha et al., 2014).

**Figure 2:**
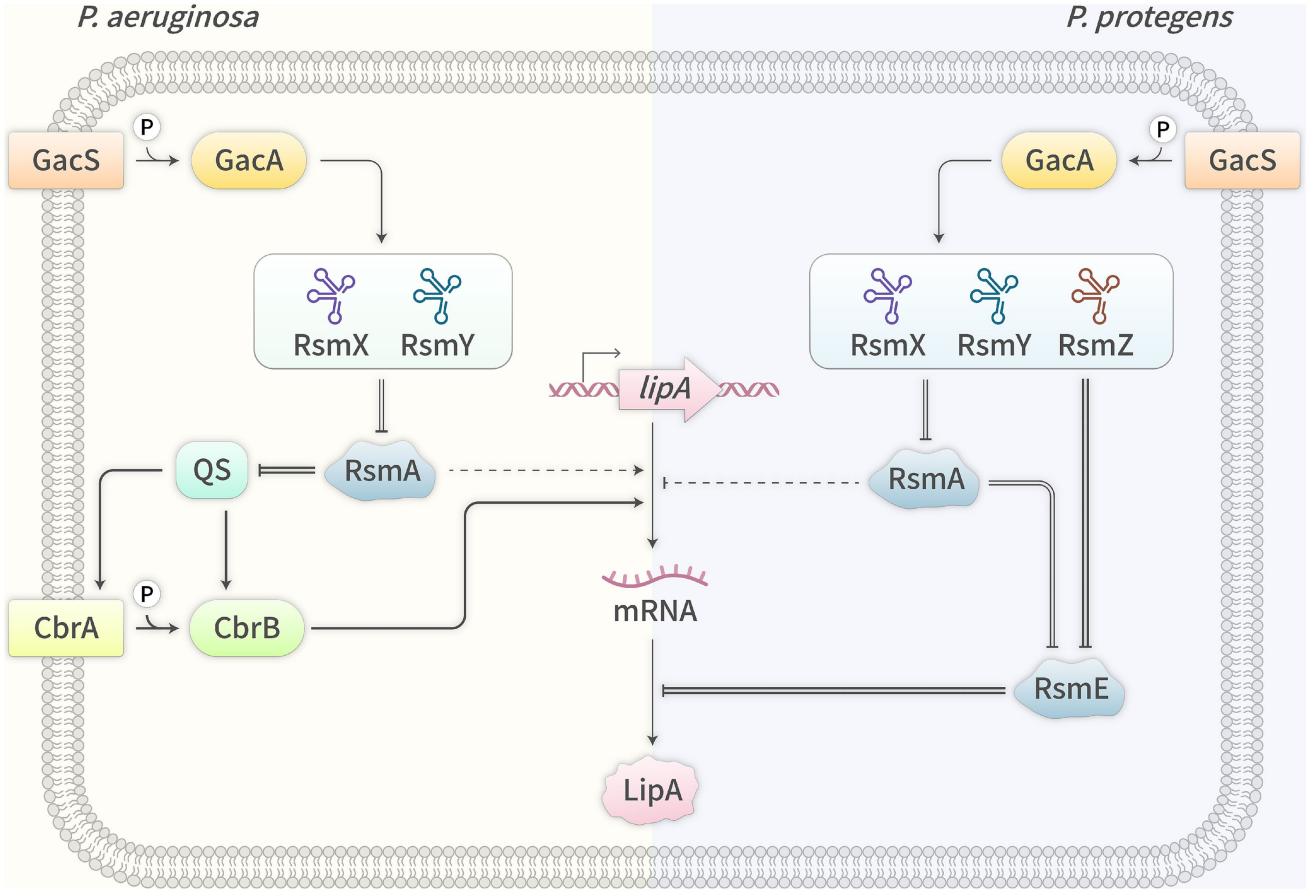
Expression regulation of lipase genes by Gac/Rsm system in *P. aeruginosa* (L) and *P. protegens* (R). →, activation; —|, inhibition; =, interaction; —, known mechanism; ┄, unknown mechanism.

In summary, in *P. aeruginosa*, the Gac/Rsm system exhibits duality in regulating *lipA* expression, primarily promoting *lipA* transcription through the Gac/RsmA/QS/CbrA/B/LipA pathway, and secondarily inhibiting *lipA* transcription through the Gac/RsmA/unknown regulator(s)/LipA pathway (Figure 2, L). However, in *P. protegens*, the system regulates *lipA* expression through other pathways, which primarily and directly activates *lipA* translation through the Gac/RsmE/LipA pathway, and secondarily and indirectly activates *lipA* transcription through the Gac/RsmA/unknown regulator(s)/LipA pathway (Figure 2, R). *P. protegens* chooses different regulatory pathways to activate *lipA* expression by the Gac/Rsm system, which may be due to the direct binding of RsmE to the SD sequence of *lipA* mRNA and this bacterium without the classical QS system.

## 5 Regulators mediated by Gac/Rsm system

Each component of the Gac/Rsm system is finely controlled by multiple regulators to adapt to complex and diverse environments (Lapouge et al., 2008). These regulators may modulate the expression of lipase genes mediated by the Gac/Rsm system. In *P. protegens*, AlgR mainly promotes *lipA* transcription by directly binding to the promoter sequence of *rsmZ*, but the mechanism of RsmZ regulating *lipA* expression is unknown. Besides, AlgR also enhances *lipA* expression at translational level with an unknown mechanism (Li et al., 2017). Also in this bacterium, Hfq mainly strengthens *lipA* translation through increasing the transcript of *rsmY* by an unknown mechanism and enhancing the stability of RmsY by directly binding to the sequence of RsmY, but the mechanism by which RsmY regulates *lipA* expression remains unclear. Moreover, Hfq also boosts *lipA* transcription via an unknown mechanism (Liu et al., 2017). In *P. aeruginosa*, the two-component system PmrB/A mainly activates *lipA* translation through the PmrB/A/RsmY/RsmA/LipA pathway by adopting the mechanism of PmrA directly binding to the promoter sequence of *rsmY*. Furthermore, PmrB/A also promotes *lipA* expression at the transcriptional level through an unknown mechanism (Liu et al., 2018). It is worth noting that the Gac/Rsm system is also controlled by other regulators, such as RetS, LadS, PA1611, SuhB, Lon, HptB, PsrA, IHF, MvaT/U, BfiS/R, CafA, GidA and TrmE (Zha and Yan, 2015), suggesting that they may also regulate the expression of lipase genes through the pathway mediated by the Gac/Rsm system or other pathways.

## 6 Other regulators

In *Staphylococcus aureus*, lipase production is repressed by the alternative sigma factor σ^B^ (Kullik et al., 1998) and enhanced by the two-component system AgrC/A (McNamara and Iandolo, 1998), but their regulatory mechanisms are still unknown. In *P. fluorescens*, the two-component system EnvZ/OmpR inhibits *lipA* expression, and the mutant of *envZ::Tn5* increases lipase production by 2-4-fold and the overexpression of *envZ* depresses lipase production by 10-20-fold. Of note, EnvZ/OmpR synergistically inhibits lipase production with NaCl, and NaCl acts through a mechanism independent of EnvZ/OmpR, but their regulatory mechanisms are also unclear (McCarthy et al., 2004). It is encouraging to note that the mechanism by which EnvZ/OmpR regulates lipase gene expression in *X. nematophila* has been elucidated, it represses *xlpA* transcription through the EnvZ/OmpR/FlhDC/FliA/XlpA pathway (Park and Forst, 2006). In *P. aeruginosa*, *lipA* expression is activated by the dimethylglycine demethylase DgcA and suppressed by the AraC/XylS family transcriptional regulator GbdR being necessary for choline catabolism, but their regulatory mechanisms remain unclear (Hampel et al., 2014). It is worth mentioning that the QS system is also controlled by other regulators in addition to the Gac/Rsm system, these regulators control the QS system either by affecting the activity or expression of signal receptors or by influencing the production of signal molecules (Juhas et al., 2005; Schuster and Greenberg, 2006; Boyer and Wisniewski-Dye, 2009; Williams and Cámara, 2009; Zha and Yan, 2015), ultimately, may regulate the expression of lipase genes.

Moreover, the expression of bacterial lipase genes is also regulated by various physiological factors, such as carbon sources, nitrogen sources, fatty acyl esters, iron, temperature, etc. Among carbon sources, organic acids such as pyruvate and succinate enhance lipase production, whereas most sugars, especially glucose, inhibit lipase production in *P. fluorescens* (Makhzoum et al., 1995). The inhibition of lipase production by most sugars may occur because lipase gene expression is subject to catabolite repression mediated by the CRP-cAMP complex. In the case of nitrogen sources, amino acids (e.g., arginine, threonine and lysine) and inorganic nitrogen sources (e.g., ammonium salts of mineral acids) support good lipase production in *P. fluorescens* (Makhzoum et al., 1995). In *Acinetobacter calcoaceticus*, casamino-acids and tryptone improve lipase yield, and the addition of ammonium further increases it. The increase in yield caused by nitrogen sources is unrelated to *lipA* transcription, suggesting that post-transcriptional processes, including enzyme protection, inactivation and secretion, must be considered important factors affecting lipase production (Cordenons et al., 1996). However, the transcription of *lipA* is repressed by high amino acid concentrations in *Bacillus subtilis* (Eggert et al., 2003). Among fatty acyl esters, many triglycerides and Spans and Tweens are strong inducers for lipase production in *P. aeruginosa*, *P. fluorescens* and *Thermus thermophilus* (Gilbert et al., 1991; Makhzoum et al., 1995; Deive et al., 2009), but triolein is a strong inhibitor in *P. fluorescens* (Makhzoum et al., 1995). Interestingly, the hydrolysis products of lipase, long-chain fatty acids like oleic acid, significantly repress lipase production in *P. aeruginosa*, *P. fluorescens* and *A. calcoaceticus* (Gilbert et al., 1991; Makhzoum et al., 1995; Kok et al., 1996), but a short-chain fatty acid, caproic acid, enhances considerably lipase production in *P. fluorescens* (Makhzoum et al., 1995). The feedback inhibition of long-chain fatty acids implies the involvement of a fatty acyl-responsive DNA-binding protein that, upon fatty acid binding, acts as a transcriptional repressor to downregulate the expression of lipase genes (Kok et al., 1996). In *P. fluorescens*, iron is found to strongly repress *lipA* transcription, this repression may be mediated by the iron-sensing Fur repressor (Woods et al., 2001). In some psychrotrophic *P. fluorescens* stains, temperature has also been reported to regulate the expression of lipase genes, with the maximum lipase production occurring below the optimal growth temperature, suggesting a cold-adaptation strategy (Merieau et al., 1993; Guillou et al., 1995; Makhzoum et al., 1995; Woods et al., 2001). The low-temperature regulation may be achieved at the post-transcriptional or post-translational level (Woods et al., 2001). All in all, the mechanisms by which these physiological factors regulate the expression of lipase genes remain unclear, but these results indicate that there are still many unidentified regulators in the expression regulatory network of lipase genes.

## 7 Conclusion and perspectives

Although some progress has been made in the expression regulation of bacterial lipase genes, there are still some challenges to be faced. For instance, there are difficulties in efficiently screening and identifying key regulators, constructing genetically engineered strains for stable and efficient homologous expression of lipases, and optimizing fermentation processes to enhance lipases production. Fortunately, the continuous development of biotechnology and the application of novel technologies have presented new opportunities for the research on the expression regulation of bacterial lipase genes. For example, synthetic biology can be leveraged to construct bacterial lipases with entirely new functions, new gene editing technologies like CRISPR allow for precise modification and optimization of bacterial lipase genes, and artificial intelligence and big data analytics based on multi-omics can be utilized to predict and dissect the complex networks involved in the expression regulation of bacterial lipase genes. In summary, researches on the expression regulation of bacterial lipase genes hold vast prospects and significant applied value. Future researches will continue to propel advancements in this field, offering more possibilities for the production and application of industrial lipases.
